# Supplementary material for: A Comparative Analysis of Two Automated Quantification Methods for Regional Cerebral Amyloid Retention: PET-Only and PET-and-MRI-Based Methods
Source: Int J Mol Sci. 2024 Jul 12;25(14):7649. doi: 10.3390/ijms25147649 (PMC11276670; doi:10.3390/ijms25147649)
Supplement: Supplementary file 1 [file ijms-25-07649-s001.zip › ijms-3083762-supplementary.pdf]

Supplementary Materials for "A comparative analysis of two automated quantification methods for regional cerebral amyloid retention: PET-only and PET-and-MRI-based methods" by Kim et al.

Supplementary Methods

Amyloid positron emission topography

Amyloid-positivity prediction models

Supplementary Tables

Table S1. The comparison of global and regional SUVRs between deep learning-based PET-MRI (SCALE PET) and PET-only (Syngo.via) methods.

Table S2. Root-mean-squared error (RMSE) between deep learning-based PET-MRI (SCALE PET) and PET-only (Syngo.via) methods for global and regional SUVRs.

Table S3. Predicting performance for amyloid positivity using global SUVR.

Supplementary Figures

Figure S1. Bland–Altman plot comparing regional SUVRs from the deep learning-based PET-MRI (SCALE PET) and the PET-only (Syngo.via) methods.

Figure S2. Comparison of receiver operating characteristic curves for predicting amyloid positivity with support vector machine (SVM) models and regional SUVRs from the deep learning-based PET-MRI (SCALE PET) and the PET-only (Syngo.via) methods in all participants group (A) and cognitively unimpaired (B), mild cognitive impairment (C), and dementia (D) subgroups.

Figure S3. Detailed imaging acquisition process.

Supplementary References

Supplementary Methods

Amyloid positron emission topography

Amyloid positron emission topography (PET) is an imaging technique used to assess the abnormal deposition of β-amyloid (Aβ) in the brain. The PET scanner detects the level of absorbed radioactive ligands in living cells or tissues, which is intravenously administered to the subject before scanning. This imaging technique has a beneficial characteristic in that it enables molecule-specific in vivo visualization in humans or animals. To identify the core pathology of Alzheimer's disease (AD), Aβ-specific ligands should be used. The first radioactive ligand was the ^{11}C-labeled Pittsburgh compound B (^{11}C-PiB) [1]. Its performance of predicting amyloid-positivity was confirmed by the autopsy study but its clinical use was limited due to the short half-life of the ^{11}C radioisotope, which is only 20 minutes. To overcome this drawback, several fluorime-18(^{18}F)-derived tracers with a longer half-life (110 minutes) were developed.

Currently, three ^{18}F amyloid PET tracers are currently available for clinical application and have been validated as the gold standard: ^{18}F-florbetapir (Amyvid™; Avid Radiopharmaceuticals), ^{18}F-florbetapir (Amyvid™; Avid Radiopharmaceuticals; approved in 2012), ^{18}F-flutemetamol (Vizamyl™; GE Healthcare; approved in 2013), and ^{18}F-florbetaben (Neuraceq™; Life Molecular Imaging; approved in 2014) [2]. Each molecule has a distinct chemical structure, resulting in different pharmacokinetic and binding properties. All three tracers have been granted by the Food and Drug Administration (FDA) and European Medicines Authority (EMA) for clinical use and they also received local regulatory approval in South Korea. In this study, we used ^{18}F-flutemetamol as routine clinical practice.

Amyloid-positivity prediction models

The logistic regression (LR) and support vector machine (SVM) models were implemented to predict visual readings of amyloid positron emission tomography (PET) assessed by nuclear medicine experts. The LR model is a prediction model that uses a linear combination of the Standardized Uptake Value Ratios (SUVRs) and applies the logit function to calculate the probability of belonging to an amyloid positive or negative group. During the fitting process, the model determines the coefficients of linear combination to minimize prediction errors [3]. The SVM model, on the other hand, finds a separating hyper-plane with a maximum margin between groups to classify the subjects during the fitting process [4].

Because of their transparency and simplicity, these classic supervised machine learning algorithms have been widely used for a variety of classification tasks and have shown reliable and robust discriminability. To test the reproducibility and model independence of the LR models' ability to discriminate between amyloid-positive and negative subjects, we additionally implemented the linear SVM model (with default model parameters specified in the scikit-learn Python library) for the same task described in the method section of the main manuscript.

Supplementary Tables

Table S3. The comparison of global and regional SUVRs deep learning-based PET-MRI (SCALE PET) and PET-only (Syngo.via) methods.

ROI	*Group*	*Syngo.via*	*SCALE PET*	*Statistics*
Global	All	0.625 ± 0.135	0.599 ± 0.163	t=4.183 (p<0.001***)
Global	CU	0.574 ± 0.108	0.532 ± 0.121	t=5.270 (p<0.001***)
Global	MCI	0.641 ± 0.137	0.620 ± 0.166	t=2.371 (p=0.018*)
Global	DE	0.703 ± 0.144	0.700 ± 0.179	t=0.128 (p=0.898)
Frontal	All	0.528 ± 0.148	0.594 ± 0.173	t=-9.968 (p<0.001***)
Frontal	CU	0.476 ± 0.116	0.525 ± 0.131	t=-5.683 (p<0.001***)
Frontal	MCI	0.545 ± 0.150	0.616 ± 0.175	t=-7.581 (p<0.001***)
Frontal	DE	0.604 ± 0.171	0.699 ± 0.192	t=-4.590 (p<0.001***)
Temporal	All	0.585 ± 0.128	0.560 ± 0.144	t=4.453 (p<0.001***)
Temporal	CU	0.539 ± 0.095	0.506 ± 0.106	t=4.770 (p<0.001***)
Temporal	MCI	0.599 ± 0.132	0.576 ± 0.149	t=2.762 (p=0.006**)
Temporal	DE	0.655 ± 0.148	0.642 ± 0.161	t=0.752 (p=0.453)
Parietal	All	0.519 ± 0.163	0.605 ± 0.169	t=-22.016 (p<0.001***)
Parietal	CU	0.447 ± 0.117	0.533 ± 0.124	t=-15.771 (p<0.001***)
Parietal	MCI	0.545 ± 0.166	0.629 ± 0.173	t=-15.783 (p<0.001***)
Parietal	DE	0.616 ± 0.177	0.710 ± 0.181	t=-8.977 (p<0.001***)
Cingulate	All	0.651 ± 0.154	0.640 ± 0.178	t=1.717 (p=0.086)
Cingulate	CU	0.590 ± 0.120	0.565 ± 0.133	t=2.824 (p=0.005**)
Cingulate	MCI	0.672 ± 0.156	0.663 ± 0.180	t=0.904 (p=0.366)
Cingulate	DE	0.739 ± 0.169	0.751 ± 0.195	t=-0.559 (p=0.576)
Striatum	All	0.665 ± 0.136	0.668 ± 0.139	t=-0.604 (p=0.546)
Striatum	CU	0.635 ± 0.114	0.615 ± 0.097	t=2.745 (p=0.006**)
Striatum	MCI	0.672 ± 0.141	0.682 ± 0.142	t=-1.246 (p=0.213)
Striatum	DE	0.721 ± 0.148	0.763 ± 0.162	t=-2.340 (p=0.020*)

Abbreviations. CU, cognitively unimpaired; MCI, mild cognitive impairment; DE, dementia.

* denotes p<0.05, ** denotes p<0.01, and *** denotes p<0.001

Table S4. Root-mean-squared error (RMSE) between deep learning-based PET-MRI (SCALE PET) and PET-only (Syngo.via) methods for global and regional SUVRs.

Group	*Global*	*Frontal*	*Temporal*	*Parietal*	*Cingulate*	*Striatum*
All	0.056 ± 0.037	0.068 ± 0.054	0.035 ± 0.026	0.088 ± 0.050	0.041 ± 0.031	0.057 ± 0.053
CU	0.061 ± 0.033	0.050 ± 0.042	0.037 ± 0.021	0.086 ± 0.041	0.040 ± 0.029	0.056 ± 0.036
MCI	0.053 ± 0.037	0.073 ± 0.055	0.034 ± 0.027	0.087 ± 0.052	0.040 ± 0.031	0.057 ± 0.056
DE	0.054 ± 0.040	0.097 ± 0.063	0.033 ± 0.029	0.099 ± 0.062	0.044 ± 0.036	0.063 ± 0.073

Abbreviations. CU, cognitively unimpaired; MCI, mild cognitive impairment; DE, dementia.

Table S3. Amyloid positivity predicting the performance of logistic regression models using global SUVR from the deep learning-based PET-MRI (SCALE PET) and the PET-only (Syngo.via) methods.

	Method	*Cut-off*	*Accuracy*	*Sensitivity*	*Specificity*	*F1 score*	*AUROC*
All	SCALE PET	0.548	0.913	0.907	0.917	0.903	0.957
	Syngo.*via*	0.631	0.888	0.811	0.951	0.867	0.935
CU	SCALE PET	0.554	0.927	0.843	0.955	0.854	0.951
	Syngo.*via*	0.609	0.877	0.741	0.924	0.755	0.906
MCI	SCALE PET	0.551	0.899	0.906	0.892	0.902	0.950
	Syngo.*via*	0.638	0.874	0.813	0.939	0.869	0.931
DE	SCALE PET	0.553	0.922	0.946	0.857	0.946	0.950
	Syngo.*via*	0.635	0.915	0.910	0.929	0.940	0.963

Supplementary Figures

Figure S1. Bland–Altman plot comparing regional SUVRs from the deep learning-based PET-MRI (SCALE PET) and the PET-only (Syngo.via) methods. The differences in global (A) and regional SUVR for (B) frontal, (C) temporal, (D) parietal, (E) cingulate, and (F) striatal areas of the two methods according to the average are displayed.

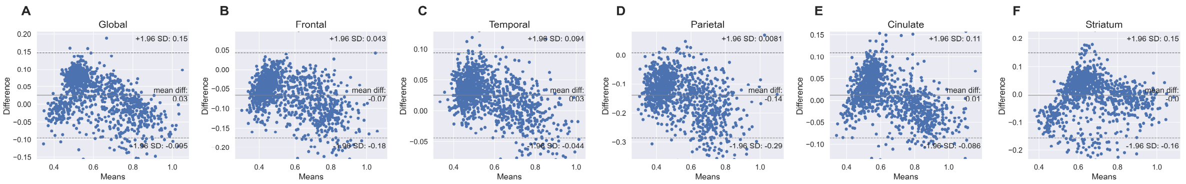

Figure S2. Comparison of receiver operating characteristics curves for predicting amyloid positivity with support vector machine (SVM) models and regional SUVRs from the deep learning-based PET-MRI (SCALE PET) and the PET-only (Syngo.via) methods in all participants group (A) and cognitively unimpaired (B), mild cognitive impairment (C), and dementia (D) subgroups.

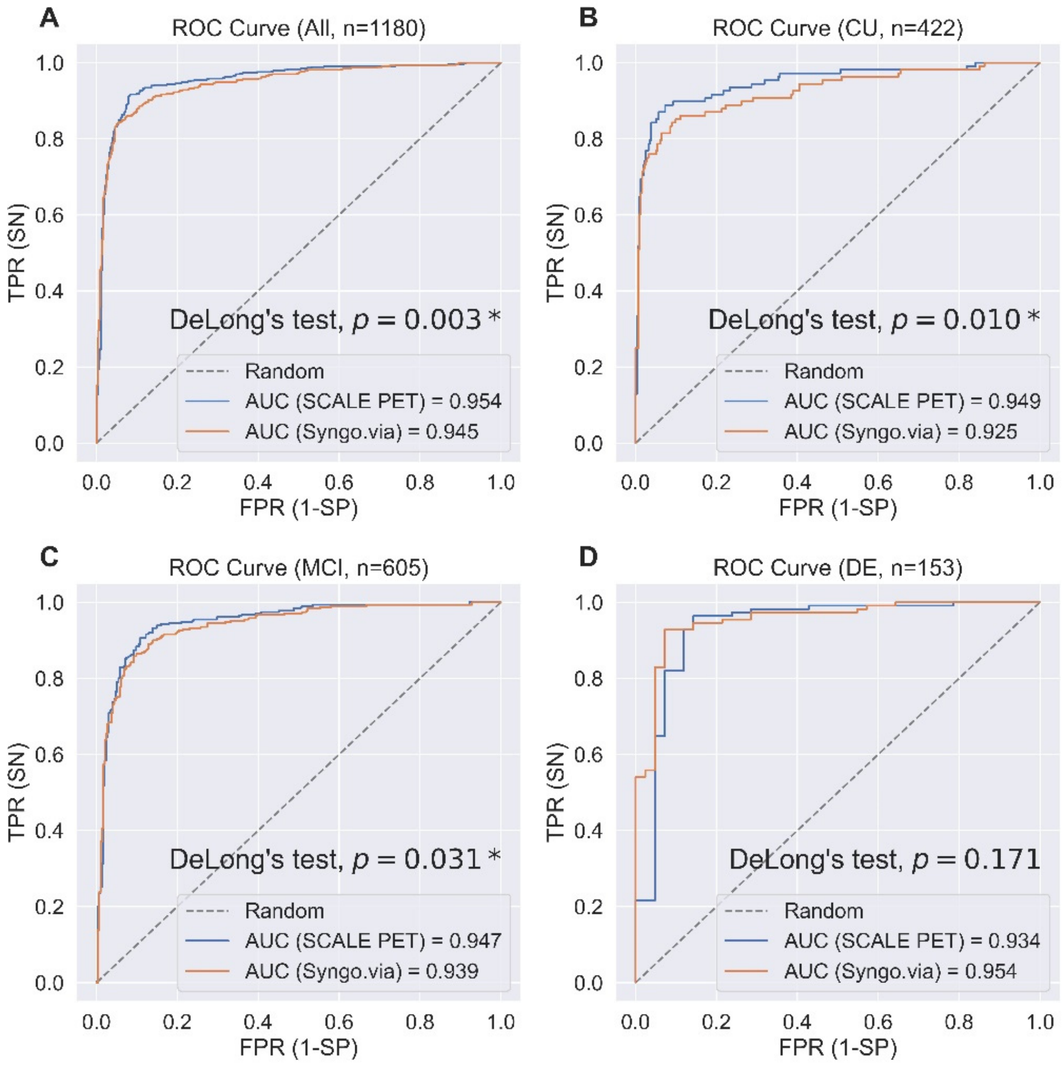

Figure S3. Detailed imaging acquisition process.

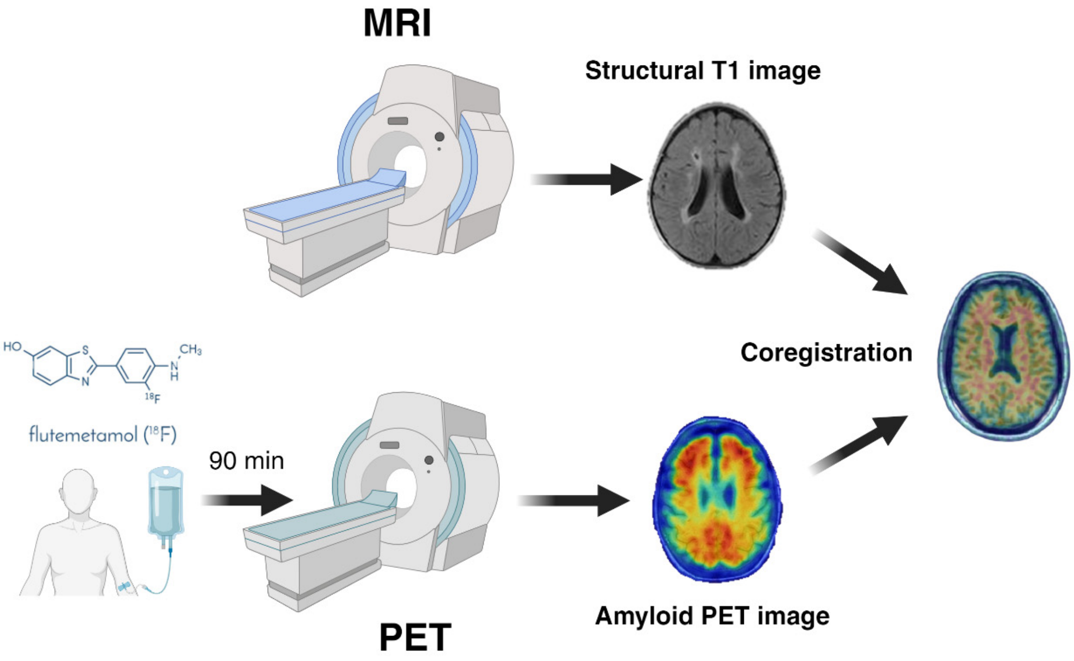

Supplementary References

[1] Klunk, W.E., et al., *Imaging brain amyloid in Alzheimer's disease with Pittsburgh Compound-B.* Annals of Neurology: Official Journal of the American Neurological Association and the Child Neurology Society, 2004. **55**(3): p. 306-319.

[2] Villemagne, V.L., et al., *Imaging tau and amyloid-β proteinopathies in Alzheimer disease and other conditions.* Nature Reviews Neurology, 2018. **14**(4): p. 225-236.

[3] Hosmer Jr, D.W., S. Lemeshow, and R.X. Sturdivant, *Applied logistic regression*. 2013: John Wiley & Sons.

[4] Vapnik, V.N. *The support vector method*. in *International conference on artificial neural networks*. 1997. Springer.
